# Risk of thromboembolic diseases in men with prostate cancer: results from the population-based PCBaSe Sweden

**DOI:** 10.1016/S1470-2045(10)70038-3

**Published:** 2010-05-11

**Authors:** Mieke Van Hemelrijck, Jan Adolfsson, Hans Garmo, Anna Bill-Axelson, Ola Bratt, Erik Ingelsson, Mats Lambe, Pär Stattin, Lars Holmberg

**Affiliations:** a King's College London, School of Medicine, Division of Cancer Studies, Cancer Epidemiology Group, London, UK; b Oncological Centre, CLINTEC Department, Karolinska Institutet, Stockholm, Sweden; c Regional Oncologic Centre, Uppsala University, Uppsala, Sweden; d Department of Urology, Uppsala University, Uppsala, Sweden; e Department of Clinical Cancer Epidemiology, Karolinska Institutet, Stockholm, Sweden; f Department of Urology, Helsingborg Hospital, Lund University, Sweden; g Department of Medical Epidemiology and Biostatistics, Karolinska Institutet, Stockholm, Sweden; h Department of Surgical and Perioperative Sciences, Urology and Andrology, Umeå University, Umeå, Sweden

## Abstract

**Background:**

Cancer is associated with an increased risk of thromboembolic diseases, but data on the association between prostate cancer and thromboembolic diseases are scarce. We investigated the risk of thromboembolic disease in men with prostate cancer who were receiving endocrine treatment, curative treatment, or surveillance.

**Methods:**

We analysed data from PCBaSe Sweden, a database based on the National Prostate Cancer Register, which covers over 96% of prostate cancer cases in Sweden. Standardised incidence ratios (SIR) of deep-venous thrombosis (DVT), pulmonary embolism, and arterial embolism were calculated by comparing observed and expected (using the total Swedish male population) occurrences of thromboembolic disease, taking into account age, calendar-time, number of thromboembolic diseases, and time since previous thromboembolic disease.

**Findings:**

Between Jan 1, 1997, and Dec 31, 2007, 30 642 men received primary endocrine therapy, 26 432 curative treatment, and 19 526 surveillance. 1881 developed a thromboembolic disease. For men on endocrine therapy, risks for DVT (SIR 2·48, 95% CI 2·25–2·73) and pulmonary embolism (1·95, 1·81–2·15) were increased, although this was not the case for arterial embolism (1·00, 0·82–1·20). Similar patterns were seen for men who received curative treatment (DVT: 1·73, 1·47–2·01; pulmonary embolism: 2·03, 1·79–2·30; arterial embolism: 0·95, 0·69–1·27) and men who were on surveillance (DVT: 1·27, 1·08–1·47; pulmonary embolism: 1·57, 1·38–1·78; arterial embolism: 1·08, 0·87–1·33). Increased risks for thromboembolic disease were maintained when patients were stratified by age and tumour stage.

**Interpretation:**

All men with prostate cancer were at higher risk of thromboembolic diseases, with the highest risk for those on endocrine therapy. Our results indicate that prostate cancer itself, prostate cancer treatments, and selection mechanisms all contribute to increased risk of thromboembolic disease. Thromboembolic disease should be a concern when managing patients with prostate cancer.

**Funding:**

Swedish Research Council, Stockholm Cancer Society, and Cancer Research UK.

## Introduction

Cancer is a risk factor for thromboembolic disease, and patients with cancer are estimated to be around four times more likely to develop a thrombosis than a similar individual without cancer.^1^ Treatments for cancer might also increase the risk of thromboembolic disease. For prostate cancer, deep-venous thrombosis (DVT) and thromboembolism are common complications after prostatectomy, with risks ranging from 0·5% to 40% in the 30 days after the operation.^2^, ^3^, ^4^, ^5^ Additionally, the risk of thromboembolic disease increases exponentially with age.^6^ While several studies have investigated whether patients with prostate cancer treated with endocrine treatment are at higher risk for cardiovascular disease, few population-based studies have investigated the risk of thromboembolic disease following endocrine treatment.^7^, ^8^, ^9^ During the 1980s, Varenhorst and colleagues^8^, ^9^ reported a positive association between the use of cyproterone acetate (a steroidal anti-androgen) and fibrinolytic activity, suggesting a decreased risk of thromboembolism. A more recent study, including 11 199 men with prostate cancer, of whom 229 had a venous thromboembolism after diagnosis, showed a greater risk of venous thrombosis associated with cyproterone acetate than with gonadrotropin releasing-hormone (GNRH) agonists or orchiectomy.^7^

Investigation of the risk of thromboembolic disease after endocrine treatment is important for several reasons. Testosterone is thought to have a cardioprotective effect, since androgen receptors have been identified on the cardiomyocytes and the valves of the heart.^10^, ^11^, ^12^, ^13^, ^14^, ^15^ Preliminary experimental findings have suggested that androgens might have a role in the regulation of arterial thrombosis through their effect on platelet activation.^16^ Endocrine treatment is used in a large proportion of men with prostate cancer during the course of the disease, and is the cornerstone treatment for men with locally advanced or metastatic prostate cancer.^17^, ^18^ The indications for endocrine treatment have been widening because of more active treatment in men with advanced disease, and because of neoadjuvant and adjuvant use in men with localised high-risk tumours, resulting in more men of all ages and with all types of prostate tumour receiving endocrine treatment for longer periods.^19^

We studied a comprehensive population-based cohort with complete follow-up through record linkage of pertinent registers, to assess the risk of thromboembolic disease in men with prostate cancer who had received curative treatment, surveillance, or endocrine treatment.

## Methods

### Data collection

PCBaSe Sweden is based on the National Prostate Cancer Register (NPCR) of Sweden, which started in 1996 and captures more than 96% of all newly diagnosed, biopsy-confirmed prostate cancers. This compares favourably with the Swedish Cancer Registry,^20^ which misses less than 3·7% of prostate cancer cases.^21^ The NPCR includes date of diagnosis, age at diagnosis, tumour stage, tumour differentiation, serum concentration of prostate cancer-specific antigen (PSA) at the time of diagnosis, and primary treatment given or planned up to 6 months after the date of diagnosis. The validity of primary treatment registered in NPCR is more than 90% for curative treatment and surveillance, and more than 95% for endocrine treatment (Stattin P, unpublished). A more detailed description of NPCR is given elsewhere.^22^

Patients had been treated in accordance with regional clinical care guidelines. Because of PSA screening, the rate of curative treatment increased dramatically during the period of study, whereas the rate of primary endocrine treatment was more or less constant over the same period. The proportion of men with localised disease put on surveillance decreased during this time.^22^ Most endocrine treatment was with GNRH agonists or orchiectomy, although anti-androgens (not combined with other endocrine treatment) were prescribed in about 10% of cases. Maximum androgen blockade was never recommended in the guidelines. Most anti-androgens used during the registration period were non-steroidal—ie, flutamide and bicalutamide. Cyproterone acetate was not recommended as a primary endocrine treatment, and was only used as second-line treatment in selected cases. For hormone-resistant prostate cancers, oestrogens and estramustine phosphate were prescribed most often.^22^

By using the Swedish 10-digit personal identity number, PCBaSe was linked to other national registers, allowing for information on demographics, comorbidities, socioeconomic status, and causes of death to be collected.^23^, ^24^ In 1987, the Hospital Discharge Register started collecting information regarding in-patient care. Each record contains medical information on surgical and anaesthetic procedures, hospital department, and discharge diagnoses coded according to the WHO International Classification of Diseases 10 (ICD10).^23^ For heart diseases, the primary diagnoses have been shown to be correct in around 95% of cases, as judged by the European Society of Cardiology diagnosis guidelines.^25^, ^26^, ^27^ Socioeconomic characteristics were assessed by record linkages to the 1960–90 5-yearly census databases, and based on socioeconomic status. Socioeconomic status is based on occupational group, and stratifies men into white-collar worker (salaried professionals or educated workers who perform a semi-professional office, administrative, or sales coordination), blue-collar worker (manual workers), not gainfully employed, and unknown.^24^ As of 1997, the Cause of Death Register collected date and underlying cause of death coded according to ICD10.^23^ Detailed information on the data content of PCBaSe is given elsewhere.^28^

For our analyses, the following information was taken from PCBaSe: age, serum concentrations of PSA, and treatment information at time of diagnosis, tumour grade and stage, socioeconomic status, history of thromboembolic disease (primary diagnoses), and date of death. Gleason score was used to assess tumour grade. If WHO tumour grade was reported primarily instead of Gleason score (25% of men), conversion to Gleason score was done as follows: G1=Gleason 2–6, G2=Gleason 7, and G3=Gleason 8–10. Prostate cancer stage was defined based on the Tumour, Node, Metastasis (TNM) stages used in the NPCR (panel).^22^ Men with prostate cancer were selected if they received curative treatment, surveillance, or endocrine treatment as primary treatment. The curative treatment group consisted of men who underwent radical prostatectomy and/or radiotherapy. Endocrine treatment was grouped into anti-androgens, oestrogens, orchiectomy, GNRH agonists, GNRH agonist combined with long-term anti-androgens, and other types of endocrine treatment.

Panel **Prostate cancer stage grouping in the National Prostate Cancer Register (NPCR) of Sweden**

1 Localised (prostate-specific antigen [PSA] <20 ng/mL)—T0–2, N0 or NX, M0 or MX, all grades, PSA <20 ng/mL
2 Localised (PSA ≥20 ng/mL but <50 ng/mL): T0–2, N0 or NX, M0 or MX, all grades, PSA ≥20 ng/mL but <50 ng/mL
3 Locally advanced—T3–4, N0 or NX, M0 or MX, all grades, PSA <50 ng/mL
4 Intermediate group—M0 or MX, PSA ≤100 ng/mL, not in stage group 1, 2, or 3
5 Metastatic disease—M1 or PSA >100 ng/mL
6 Missing data—Missing T or N or M category/categories or missing grade or missing PSA

The Swedish central ethics committee (Dnr Ö 14-2007) and the ethics committee at Umeå University (Dnr 07-049M) approved this study.

### Statistical analysis

We analysed the relation between the different types of prostate cancer treatment and subtypes of thromboembolic disease: DVT (ICD10: I80–82), pulmonary embolism (ICD10: I26), and arterial embolism (ICD10: K55, I74). Since PCBaSe is based on the entire Swedish population, standardised incidence ratios (SIR) could be calculated by comparing observed events in the selected cohort (men with prostate cancer) with the expected events in the Swedish male population. The number of events for this standard population was based on the number of men in Sweden each year on Dec 31 (Register of the Total Population 1997–2007).^23^, ^24^ All numbers of events were based on the first event of thromboembolic disease after a diagnosis of prostate cancer.

The SIRs were thus defined as the ratio of the observed number of a particular thromboembolic disease to the expected number of that thromboembolic disease. The calculations of these SIRs are explained in detail in the webappendix. Briefly, observed numbers of thromboembolic disease were counted among the men with prostate cancer, and were obtained from the Hospital Discharge Register (1987–2007). Expected numbers were calculated by multiplying time of follow-up with the corresponding age-specific and period-specific incidence rates. Time of follow-up was taken from the observed numbers of thromboembolic disease, and age-specific and period-specific incidence rates were calculated as the incidence of thromboembolic disease in the background population divided by the corresponding person-time in this background population. All calculations took thromboembolic disease history into account, since men with a history of previous thromboembolism are at an increased risk for being diagnosed with prostate cancer or a subsequent thromboembolic disease.^29^ The 95% CI for the SIRs were estimated by assuming that the observed cases had a Poisson distribution using Byar's normal approximation.^30^, ^31^ To take comorbidities into account before prostate cancer diagnosis, SIR calculations were also stratified by history of ischaemic heart disease (ICD10: I20-25), circulatory disease (ICD10: I00-I99), and stroke (ICD10: I60–64, G45). All analyses were stratified by tumour stage and age group (<65, 65–74, and ≥75 years). Poisson regression was used to adjust the SIRs for cancer stage, history of thromboembolic disease, and socioeconomic status. The absolute risk differences by different types of thromboembolic disease and prostate cancer treatment were calculated, and sensitivity analyses were done to test the assumption of intention-to-treat. Finally, we calculated the bias in the SIRs due to using the general population rates to estimate the expected numbers of thromboembolic disease, based on the formulas by Jones and Swerdlow.^32^ Statistical analyses were done with SAS version 9.1.3, and R version 2.7.2.

### Role of the funding source

The funding organisations had no influence on the design and conduct of the study, data collection, management, analysis, interpretation, and preparation, review, or approval of the manuscript. The corresponding author had full access to all data, and the final responsibility to submit the manuscript for publication.

## Results

Between Jan 1, 1997, and Dec 31, 2007, PCBaSe registered 76 600 men diagnosed with prostate cancer, of whom 30 642 received endocrine treatment as their primary treatment. Specifically, 3391 men received anti-androgen therapy; 5340 underwent orchiectomy; 9066 received a GNRH agonist; and 11 646 men received GNRH agonists combined with short-term anti-androgen therapy (table 1). The remaining 1199 men were treated with other types or combinations of endocrine treatment. The remaining 45 958 men received either curative treatment (n=26 432) or surveillance (n=19 526; table 1).

**Table 1:** Baseline characteristics of men with prostate cancer according to their treatment in PCBaSe Sweden

		**Endocrine treatment**					**Curative treatment**	**Surveillance**
		Total endocrine treatment group	Anti-androgens	Orchiectomy	GNRH agonists	GNRH agonists plus short-term anti-androgen therapy		
Total (n)		30 642*	3391	5340	9066	11 646	26 432	19 526
Mean follow-up time (SD)		3·5 (2·4)	4·0 (2·4)	3·1 (2·3)	3·8 (2·4)	3·3 (2·3)	4·4 (2·5)	4·7 (2·7)
Age group (years)								
	<65	2941 (9·6)	546 (16·1)	208 (3·9)	731 (8·1)	1140 (9·8)	13 677 (51·7)	2535 (13·0)
	65–74	9255 (30·2)	1505 (44·4)	1128 (21·1)	2706 (29·8)	3499 (30·0)	10 956 (41·4)	7526 (38·5)
	≥75	18 446 (60·2)	1340 (39·5)	4004 (75·0)	5629 (62·1)	7007 (60·2)	1799 (6·8)	9465 (48·5)
Date period								
	1997–99	8503 (27·7)	534 (15·7)	2114 (39·6)	3113 (34·3)	2316 (19·9)	3849 (14·6)	5470 (28·0)
	2000–02	9669 (31·6)	1099 (32·4)	1507 (28·2)	2848 (31·4)	3850 (33·1)	6732 (25·5)	5618 (28·8)
	2003–06	12 470 (40·7)	1758 (51·8)	1719 (32·2)	3105 (34·2)	5480 (47·1)	15 851 (60·0)	8438 (43·2)
Gleason score								
	2–6	6042 (19·7)	1016 (30·0)	851 (15·9)	2005 (22·1)	1961 (16·8)	15 325 (58·0)	13 277 (68·0)
	7	12 192 (39·8)	1438 (42·4)	2069 (38·7)	3588 (39·6)	4690 (40·3)	8153 (30·8)	4687 (24·0)
	8–10	11 562 (37·7)	877 (25·9)	2236 (41·9)	3205 (35·4)	4696 (40·3)	2729 (10·3)	1152 (5·9)
	Missing data	846 (2·8)	60 (1·8)	184 (3·4)	268 (3·0)	299 (2·6)	225 (0·9)	410 (2·1)
Prostate cancer stage group								
	Localised: PSA <20 ng/mL	4298 (14·0)	838 (24·7)	469 (8·8)	1516 (16·7)	1348 (11·6)	18 850 (71·3)	12 879 (66·0)
	Localised: PSA ≥20 ng/mL but <50 ng/mL	3317 (10·8)	516 (15·2)	460 (8·6)	1101 (12·1)	1158 (9·9)	2247 (8·5)	2411 (12·3)
	Locally advanced	5323 (17·4)	643 (19·0)	829 (15·5)	1779 (19·6)	1931 (16·6)	2600 (9·8)	1696 (8·7)
	Intermediate	4845 (15·8)	635 (18·7)	706 (13·2)	1561 (17·2)	1740 (14·9)	853 (3·2)	750 (3·8)
	Metastatic disease or PSA >100 ng/mL	12 139 (39·6)	671 (19·8)	2726 (51·0)	2845 (31·4)	5269 (45·2)	592 (2·2)	456 (2·3)
	Missing data	720 (2·3)	88 (2·6)	150 (2·8)	264 (2·9)	200 (1·7)	1290 (4·9)	1334 (6·8)
Civil status								
	Married	19 630 (64·1)	2356 (69·5)	3202 (60·0)	5768 (63·6)	7488 (64·3)	19451 (73·6)	13 379 (68·5)
	Single	10 980 (35·8)	1033 (30·5)	2128 (39·9)	3291 (36·3)	4147 (35·6)	6972 (26·4)	6118 (31·3)
	Missing data	32 (0·1)	2 (0·1)	10 (0·2)	7 (0·1)	11 (0·1)	9 (0·1)	29 (0·1)
Socioeconomic status								
	White collar	13 049 (42·6)	1749 (51·6)	1929 (36·1)	3839 (42·3)	4959 (42·6)	14 322 (54·2)	9203 (47·1)
	Blue collar	17 105 (55·8)	1592 (46·9)	3334 (62·4)	5066 (55·9)	6504 (55·8)	11 805 (44·7)	10 052 (51·5)
	Not gainfully employed/missing data	488 (1·6)	50 (1·5)	77 (1·4)	161 (1·8)	183 (1·6)	305 (1·2)	271 (1·4)
Thromboembolic disease at baseline								
	Yes	1143 (3·7)	92 (2·7)	194 (3·6)	376 (4·1)	440 (3·8)	487 (1·8)	649 (3·3)
	No	29 499 (96·3)	3299 (97·3)	5146 (96·4)	8690 (95·9)	11 206 (96·2)	25 945 (98·2)	18 877 (96·7)

* Includes 1119 men who were treated with other types or combinations of endocrine therapy. Data are n (%) unless otherwise stated. GNRH=gonadotropin-releasing hormone. PSA=prostate cancer-specific antigen. SD=standard deviation.

The characteristics of the study population are shown in table 1. 18 446 (60·2%) of the men given endocrine treatment were aged 75 years or more, compared with 9465 (48·5%) of those on surveillance, and 1799 (6·8%) of those who received curative treatment. Men treated with anti-androgens were younger on average than men treated with other types of endocrine treatment (table 1). In the endocrine treatment group, 4298 (14·0%) patients had a localised tumour and PSA concentration less than 20 ng/mL, compared with 12 879 (66·0%) in the surveillance group, and 18 850 (71·3%) in the curative treatment group. In the group treated with anti-androgens, 838 (24·7%) men had localised tumours, compared with 643 (19·0%) with locally advanced disease, and 671 (19·8%) with metastatic disease. 2845 (31·4%) men treated with GNRH agonists had metastatic disease, while 2726 (51·0%) men treated with orchiectomy had metastatic disease.

1881 men developed a thromboembolic disease after being diagnosed with prostate cancer: 767 men had a DVT, 873 a pulmonary embolism, and 241 an arterial embolism. In the total group with prostate cancer, the SIR was 1·90 (95% CI 1·77–2·04) for DVT, 1·85 (1·73–1·97) for pulmonary embolism, and 1·02 (0·89–1·15) for arterial embolism. When analysed according to prostate cancer treatment, the risks for DVT and pulmonary embolism were increased irrespective of whether patients received endocrine treatment, were treated curatively, or were on surveillance (table 2). No increased risk for arterial embolism was noted in any of the groups. Results according to prostate cancer treatment were also analysed for different strata of comorbidities before prostate cancer diagnosis, but there was no indication of effect modification by history of any circulatory disease (data not shown).

**Table 2:** Standard incidence ratios (SIR) with 95% CI for different groups of patients with prostate cancer patients in PCBaSe Sweden

	**Patients with prostate cancer on endocrine treatment**		**Patients with prostate cancer on curative treatment**		**Patients with prostate cancer on surveillance**	
	SIR (95% CI)	Obs/Exp	SIR (95% CI)	Obs/Exp	SIR (95% CI)	Obs/Exp
Deep-venous thrombosis	2·48 (2·25–2·73)	436/175·6	1·73 (1·47–2·01)	160/92·7	1·27 (1·08–1·47)	171/135·1
Pulmonary embolism	1·95 (1·81–2·15)	380/195·2	2·03 (1·79–2·30)	248/122·1	1·57 (1·38–1·78)	245/155·6
Arterial embolism	1·00 (0·82–1·20)	108/108·5	0·95 (0·69–1·27)	44/46·4	1·08 (0·87–1·33)	89/82·1

Obs=observed. Exp=expected.

Detailed analysis showed that adjustment for Gleason score or civil status did not alter the SIRs appreciably (data not shown), but different effects by age and tumour stage at time of diagnosis were found. Age-stratified and tumour-stratified analyses showed a larger SIR for men younger than 75 years, and for those with metastatic disease, in each treatment group (table 3). Overall, the SIRs for DVT were larger for men on endocrine treatment than for men who had undergone curative treatment or men who were on surveillance, but there was not such a distinct pattern for pulmonary embolism (table 3).

**Table 3:** Standard incidence ratios (SIR) for different groups of thromboembolic diseases in patients with prostate cancer according to their treatment and stratified by age and tumour stage

		**Endocrine treatment**		**Curative treatment**		**Surveillance**	
		SIR (95% CI)	Obs/Exp	SIR (95% CI)	Obs/Exp	SIR (95% CI)	Obs/Exp
**Deep-venous thrombosis**							
All tumour stages*							
	<65 years	6·18 (4·56–8·20)	48/7·8	2·22 (1·74–2·78)	74/33·4	1·07 (0·46–2·11)	8/7·5
	65–74 years	3·09 (2·61–3·63)	148/47·9	1·42 (1·11–1·80)	70/49·1	1·27 (0·97–1·64)	61/47·9
	≥75 years	2·00 (1·75–2·27)	240/120	1·57 (0·89–2·54)	16/10·2	1·28 (1·04–1·55)	102/79·8
Localised tumours							
	<65 years	2·77 (0·56–8·09)	3/1·1	2·03 (1·53–2·63)	56/27·6	0·91 (0·33–1·98)	6/6·6
	65–74 years	1·53 (0·94–2·33)	21/13·8	1·34 (0·99–1·77)	50/37·3	1·23 (0·91–1·64)	47/38·1
	≥75 years	1·66 (1·26–2·13)	60/36·2	1·03 (0·38–2·25)	6/5·8	1·17 (0·90–1·49)	66/56·4
Intermediate tumours							
	<65 years	4·78 (2·67–7·88)	15/3·1	2·48 (1·19–4·56)	10/4	2·38 (0·03–13·26)	1/0·4
	65–74 years	3·03 (2·28–3·96)	54/17·8	1·84 (1·05–2·99)	16/8·7	1·77 (0·88–3·16)	11/6·2
	≥75 years	1·96 (1·57–2·43)	85/43·3	2·56 (0·83–5·98)	5/2	1·49 (0·92–2·28)	21/14·1
Metastatic tumours							
	<65 years	8·63 (5·82–12·32)	30/3·5	14·37 (5·25–31·29)	6/0·4	16·37 (0·21–91·10)	1/0·1
	65–74 years	4·22 (3·25–5·37)	65/15·4	3·89 (0·78–11·36)	3/0·8	1·85 (0·21–6·66)	2/1·1
	≥75 years	2·34 (1·87–2·89)	86/36·8	2·41 (0·27–8·71)	2/0·8	1·28 (0·26–3·73)	3/2·3
**Pulmonary embolism**							
All tumour stages*							
	<65 years	4·92 (3·60–6·56)	46/9·4	1·93 (1·55–2·37)	90/46·7	2·22 (1·39–3·37)	22/9·9
	65–74 years	1·89 (1·54–2·29)	104/55·1	2·01 (1·68–2·39)	128/63·6	1·44 (1·14–1·78)	82/57
	≥75 years	1·76 (1·54–2·00)	230/130·7	2·53 (1·71–3·62)	30/11·8	1·59 (1·34–1·87)	141/88·7
Localised tumours							
	<65 years	1·40 (0·16–5·04)	2/1·4	1·72 (1·33–2·18)	67/39	2·03 (1·20–3·21)	18/8·9
	65–74 years	1·31 (0·82–1·99)	22/16·8	1·93 (1·56–2·36)	95/49·2	1·34 (1·03–1·72)	62/46·2
	≥75 years	1·39 (1·05–1·79)	58/41·8	2·76 (1·66–4·30)	19/6·9	1·56 (1·27–1·90)	100/64·1
Intermediate tumours							
	<65 years	4·74 (2·81–7·50)	18/3·8	3·18 (1·85–5·10)	17/5·3	6·38 (1·28–18·63)	3/0·5
	65–74 years	1·55 (1·06–2·19)	32/20·7	2·26 (1·45–3·36)	24/10·6	2·05 (1·12–3·44)	14/6·8
	≥75 years	1·76 (1·4–2·19)	83/47·1	1·86 (0·50–4·77)	4/2·1	1·13 (0·66–1·81)	17/15
Metastatic tumours							
	<65 years	6·42 (4·2–9·41)	26/4	1·88 (0·02–10·47)	1/0·5	0 (NA–53·27)	0/0·1
	65–74 years	2·95 (2·18–3·90)	49/16·6	3·47 (0·70–10·15)	3/0·9	4·82 (1·55–11·24)	5/1
	≥75 years	2·08 (1·64–2·59)	79/38·1	3·29 (0·66–9·62)	3/0·9	1·73 (0·46–4·42)	4/2·3
**Arterial embolism**							
All tumour stages*							
	<65†, 65–74†, ≥75†	1·29 (0·35–3·31)	4/3·1	0·94 (0·50–1·60)	13/13·9	0·64 (0·07–2·30)	2/3·1

NA=not available. Obs=observed. Exp=expected.

* Includes patients with missing data on stage (see panel for definitions of tumour stages).

† Not broken down by age group due to small numbers.

In age-stratified analyses for different types of endocrine treatment, the smallest SIR for both DVT and pulmonary embolism was noted for men treated with anti-androgens, whereas the largest was seen for orchiectomy (table 4). The numbers of men with arterial embolism were too small to do this subanalysis. The age effect seen for the overall endocrine treatment group in table 3 was also noted for each type of endocrine treatment, although it was less strong because endocrine treatment preferences vary by age—around 20% of the men in the two younger age groups received anti-androgens, and 9% underwent orchiectomy, whereas in men aged 75 years and over a higher proportion underwent orchiectomy (20%) and a lower proportion received anti-androgens (10%).

**Table 4:** Standard incidence ratios (SIR) for different groups of thromboembolic disease by type of endocrine treatment

	**Anti-androgens**		**Orchiectomy**		**GNRH agonists**		**GNRH agonists plus short-term anti-androgen therapy**	
	SIR (95% CI)	Obs/Exp	SIR (95% CI)	Obs/Exp	SIR (95% CI)	Obs/Exp	SIR (95% CI)	Obs/Exp
**Deep-venous thrombosis**								
All ages	1·56 (1·03–2·27)	27/17·3	2·81 (2·26–3·45)	90/32·1	2·42 (2·04–2·86)	142/58·6	2·51 (2·13–2·94)	154/61·3
<65 years*	1·14 (0·21–6·36)	..	1·76 (0·23–13·51)	..	4·03 (0·94–17·32)	..	2·32 (0·53–10·16)	..
65–74 years†	0·69 (0·27–1·73)	..	1·36 (0·54–3·42)	..	0·96 (0·41–2·23)	..	1·38 (0·60–3·17)	..
≥75 years†	0·98 (0·47–2·07)	..	1·81 (1·03–3·18)	..	1·49 (0·89–2·50)	..	1·30 (0·76–2·22)	..
**Pulmonary embolism**								
All ages	1·35 (0·91–1·94)	29/21·4	2·26 (1·77–2·84)	73/32·4	1·84 (1·52–2·20)	118/64·1	2·00 (1·68–2·36)	141/70·6
<65 years*	0·23 (0·03–1·73)	..	0·65 (0·08–5·50)	..	0·63 (0·10–4·08)	..	0·36 (0·05–2·42)	..
65–74 years†	0·46 (0·16–1·36)	..	0·77 (0·25–2·36)	..	0·83 (0·31–2·20)	..	0·82 (0·31–2·21)	..
≥75 years†	0·81 (0·39–1·67)	..	1·17 (0·62–2·18)	..	0·84 (0·47–1·51)	..	0·98 (0·54–1·77)	..

Obs=observed. Exp=expected.

* Adjusted for tumour stage, socioeconomic status, and history of thromboembolic disease.

† Adjusted for tumour stage, history of thromboembolic disease, socioeconomic status, and 5-year age groups. GNRH=gonadotropin-releasing hormone.

Sensitivity analyses excluding men who had an event of thromboembolic disease within 31 days after prostate cancer diagnosis did not alter the previous findings significantly: changes in SIRs ranged between 0% and 0·50% (data not shown). Additionally, we analysed the time between prostate cancer diagnosis and first event of thromboembolic disease (table 5). Increased risks for thromboembolic events for all treatment groups seem to be dominated by events occurring during the first 18 months after diagnosis, but an increased risk was still noted after more than 4 years. A more detailed analysis of curative treatment indicated that the risks were highest during the first 6 months, and were higher for radical prostatectomy than for radiotherapy (data not shown).

**Table 5:** Standard incidence ratios (SIR) for different groups of patients with prostate cancer in PCBaSe Sweden stratified by time since prostate cancer diagnosis

	**Endocrine treatment**		**Curative treatment**		**Surveillance**	
	SIR (95% CI)	Obs/Exp	SIR (95% CI)	Obs/Exp	SIR (95% CI)	Obs/Exp
Deep-venous thrombosis (all tumour stages*)						
0–6 months	3·13 (2·52–3·84)	91/29·1	4·10 (3·01–5·45)	47/11·5	1·68 (1·11–2·43)	28/16·7
7–18 months	2·12 (1·73–2·58)	99/46·6	1·81 (1·28–2·50)	37/20·4	1·54 (1·12–2·06)	45/29·2
1·5–4 years	2·33 (1·97–2·73)	152/65·3	1·17 (0·84–1·58)	41/35·2	1·09 (0·82–1·42)	55/50·4
>4 years	2·71 (2·19–3·32)	94/34·6	1·36 (0·95–1·90)	35/25·7	1·11 (0·80–1·49)	43/38·7
Pulmonary embolism (all tumour stages*)						
0–6 months	2·57 (2·00–3·25)	69/26·9	6·57 (5·23–8·16)	82/12·5	1·45 (0·92–2·18)	23/15·8
7–18 months	2·00 (1·62–2·44)	95/47·6	2·21 (1·66–2·88)	54/24·4	1·64 (1·21–2·17)	49/29·9
1·5–4 years	1·68 (1·40–2·01)	124/73·7	1·21 (0·92–1·57)	56/46·2	1·44 (1·15–1·79)	82/56·9
>4 years	1·96 (1·58–2·4)	92/47	1·44 (1·09–1·87)	56/39	1·72 (1·38–2·11)	91/53
Arterial embolism (all tumour stages*)						
0–6 months	0·68 (0·34–1·22)	11/16·1	1·78 (0·81–3·39)	9/5	0·58 (0·19–1·35)	5/8·6
7–18 months	1·07 (0·71–1·55)	28/26·2	1·10 (0·53–2·02)	10/9·1	0·70 (0·35–1·25)	11/15·7
1·5–4 years	1·00 (0·72–1·35)	41/41·1	0·75 (0·40–1·29)	13/17·2	NA	0/0
>4 years	1·11 (0·74–1·61)	28/25·1	0·80 (0·41–1·40)	12/15	1·57 (1·13–2·11)	43/27·4

* Includes patients with missing data on stage (see panel for definitions of tumour stages). Obs=observed. Exp=expected.

Table 6 shows the absolute risk of DVT and pulmonary embolism for different prostate cancer treatment groups. The largest absolute risks were seen for DVT and for men on endocrine treatment. For the general population, it can be seen that the absolute risk for DVT was higher among men aged 75 years and over than for those aged under 75 years (table 7); by contrast, the absolute risk for DVT in men treated with endocrine treatment was comparable for younger and older men. Therefore, the absolute risk increase after exposure to endocrine treatment was larger for those in the youngest age groups (<65 years and 65–74 years) than those in the oldest age group. The pattern was similar for pulmonary embolism (data not shown).

**Table 6:** Absolute risk and absolute risk difference for deep-venous thromboembolism and pulmonary embolism disease by prostate cancer treatment in PCBaSe Sweden

	**Absolute risk for men with prostate cancer**	**Absolute risk for men in the general population**	**Absolute risk difference (95% CI)**
**Deep-venous thrombosis**			
Endocrine treatment	4·08	1·64	2·44 (2·05–2·82)
Curative treatment	1·40	0·81	0·59 (0·37–0·80)
Surveillance	1·89	1·49	0·39 (0·11–0·68)
**Pulmonary embolism**			
Endocrine treatment	3·55	1·83	1·73 (1·37–2·09)
Curative treatment	2·17	1·07	1·10 (0·83–1·37)
Surveillance	2·70	1·72	0·99 (0·65–1·33)

**Table 7:** Absolute risk and absolute risk difference of deep-venous thrombosis by prostate cancer treatment and age group in PCBaSe Sweden

	**Absolute risk for men with prostate cancer**	**Absolute risk for men in the general population**	**Absolute risk difference (95% CI)**
**Endocrine treatment**			
<65 years	4·43	0·72	3·71 (2·46 to 4·96)
65–74 years	4·05	0·39	3·66 (3·00 to 4·31)
≥75 years	4·04	2·58	1·45 (0·94 to 1·96)
**Curative treatment**			
<65 years	1·23	0·55	0·67 (0·39 to 0·95)
65–74 years	1·44	0·61	0·83 (0·49 to 1·17)
≥75 years	2·84	5·29	−2·45 (−3·84 to −1·06)
**Surveillance**			
<65 years	0·66	0·62	0·04 (−0·41 to 0·50)
65–74 years	0·66	0·41	0·25 (−0·01 to 0·51)
≥75 years	3·40	2·77	0·64 (0·07 to 1·21)

Finally, we calculated the possible bias in the SIRs due to using general population rates to estimate expected numbers of thromboembolic disease. The true relative risk (RR) of DVT was defined as SIR×(1–Prev)/(1–(Prev×SIR)), where the prevalence (Prev) of men with prostate cancer receiving endocrine treatment was estimated to be 560/100 000.^33^ This resulted in the following bias: [(RR–SIR)/SIR]×100=[(2·50–2·48)/2·48]×100=0·84%, indicating that including men with prostate cancer in the general population only resulted in a deviation of less than 1% from the so-called true SIR estimates. The size of the bias was similar for other thromboembolic diseases and other treatment groups (data not shown).

## Discussion

In this large population-based study, we compared the risk of thromboembolic disease between Swedish men with prostate cancer and Swedish men in the background population. The findings show that men with prostate cancer are at a higher risk for thromboembolic disease than are men without prostate cancer. The risk was increased for DVT and pulmonary embolism, but not for arterial embolism, and was especially high for men treated with endocrine treatment. Additionally, the relative risk of thromboembolic disease in men treated with endocrine therapy was higher for younger men (<65 years) and for men with metastatic disease, while the absolute risk was similar for all three age groups (<65, 65–74, and ≥75 years). Moreover, a smaller increase in risk was found for men treated with anti-androgens compared with the other types of endocrine treatment.

The underlying mechanisms for the higher risk of thromboembolic disease could have several explanations. First, a baseline risk might be present because of physiological alterations due to the tumour, which seems to be supported by the fact that the risk of thromboembolic disease increases as tumour stage increases. Second, the different patterns of risk associated with different types of treatment indicate that treatments, and the selection of these treatments, can affect the risk of thromboembolic disease. Curative treatment, such as prostatectomy, and surveillance are also associated with an increased risk of thromboembolic disease, and indicate that some men might have received surveillance because of ongoing comorbidities.^34^ Third, the higher risks, through each stage of the analysis, for men primarily treated with endocrine treatment indicate a risk conferred by endocrine treatment over and above the other treatments and indications for treatment.

People with cancer have an increased risk of thromboembolic disease. Even though this association has long been recognised in clinical practice, few studies have quantified this risk for men with prostate cancer in detail.^7^, ^35^, ^36^ High rates of thrombosis have been reported in other cancers, especially in people with advanced disease receiving antitumour treatment. Clinical trials on breast cancer reported a rate of thrombosis of 1–10% in women with node-positive breast cancer, whereas development of venous thrombosis was reported in 10% of women with advanced ovarian cancer and in up to 28% of people with malignant gliomas.^36^

Treatment for prostate cancer can also be associated with an increased risk of thromboembolic disease. A cohort study based on 5951 patients undergoing prostatectomy showed an incidence of 0·5% (95% CI 0·4–0·7) for symptomatic DVT and pulmonary embolism.^35^ Additionally, a British study including 11 199 men with advanced prostate cancer showed that patients treated with cyproterone acetate had a significantly higher risk for venous thromboembolism than did men who underwent orchiectomy or were prescribed GNRH agonists (adjusted odds ratio 5·23, 95% CI 3·12–8·79).^7^

We caution that the observed contrasts in thromboembolic disease between different treatment groups should be interpreted as how treatment and treatment selection modify the risk of thromboembolic disease in men with prostate cancer. For several reasons, this study cannot directly quantify how much the observed differences in thromboembolic disease risk between treatment groups are due to the treatments themselves.^1^ Factors taken into account during the process of selecting treatment might also be associated with risk of thromboembolic disease. During the period of time covered by our study, Swedish men with early-stage prostate cancer who received curative treatment had lower all-cause mortality than the background population. Most men receiving curative treatment were recommended surgery, and had to be healthy enough to undergo radical prostatectomy.^34^ We made a similar observation in our study: men 75 years or older who had undergone curative treatment had a much lower absolute risk for DVT than men of the same age in the standard population, illustrating a selection bias towards healthy men for radiotherapy and prostatectomy. The increased risk of thromboembolism in the group treated with curative intent occurred mainly during the first 6 months of follow-up, indicating that the surgical intervention was important. However, a selection phenomenon might lead to a wrong conclusion about the effect of surgery in a direct comparison with the first period of follow-up in, for example, men under surveillance.^2^ There might have been differences in diagnostic activity (frequency of check-ups and differences in the types of testing used) for thromboembolic disease between the groups. However, DVT is likely to be correctly diagnosed and clinically relevant in patients admitted to hospital, who were the only ones included in this study. Therefore, we avoided the possibility that surveillance would lead to the diagnoses of many asymptomatic DVTs not related to the cancer or the treatment. Vigilance for thromboembolic disease is likely to have been similar in men with advanced cancer and those offered curative treatment, but might have been less intense in men under surveillance. It is also possible that a rapidly fatal pulmonary embolism in patients with advanced cancer could be interpreted as the fatal end-stage of the cancer, and therefore not coded in hospital charts. However, this misclassification would only affect a smaller number of patients, and mainly those on endocrine treatment, biasing their estimates towards null.^3^ The comparison between the treatment groups might be confounded by the introduction of second-line treatment—eg, men treated with curative intent or surveillance will have been exposed to endocrine treatment when the disease progressed.

Experimental findings have suggested a link between prostate cancer and thromboembolic disease, which might lead to hypotheses for further mechanistic studies. Babiker and colleagues^37^ showed that the early release of prostasomes from prostate cancer cells into the circulation might evoke blood-clotting effects causing thromboembolic disease. Another study by Li and colleagues^16^ showed a possible link between endocrine treatment and thromboembolic disease, in which they noted that the prevention of experimental arterial thrombosis by the use of androgens at physiological concentrations is mediated by the androgen receptor through modulation of platelet activation. Some studies have also suggested that testosterone has an antithrombotic effect, because higher concentrations are associated with an increase in antithrombin 3.^9^, ^38^, ^39^ This possible antithrombotic effect of testosterone is supported by our treatment-specific analyses of endocrine treatment, which showed that men treated with anti-androgens have the lowest SIR. Anti-androgens block the androgen receptors in the prostate, but do not decrease the circulating concentrations of total testosterone. Because of the effect of anti-androgens in the hypothalamus, the testosterone concentrations in serum might even be increased, and thus androgen-dependent pathways in other organs can still function.^40^

The higher SIRs in the youngest age group (table 3) can be explained by a lower absolute thromboembolic disease risk for younger men than for older men in the general population, and similar absolute risks for younger and older men with prostate cancer. This age effect was seen within each tumour stage; however, a stronger effect was seen for those with metastatic disease at time of diagnosis, suggesting that advanced cancer potentiates the risk due to the associated predisposition for thromboembolic disease. From a public-health point of view, both the absolute risk and absolute risk difference were largest for men given endocrine treatment. Thus, the largest number of extra cases of thromboembolic disease are likely to be seen in this patient group.

The NPCR database contains data from more than 76 000 men with prostate cancer, and provides complete follow-up for each patient, as well as linkage to other registers that allow for detailed information on thromboembolic disease morbidity. The same information about thromboembolic disease was available for the entire general population, which enabled us to adjust all comparisons for history of thromboembolic disease. Milder, non-hospitalised cases of thromboembolic disease, such as asymptomatic DVT, were not included, thus there is a possible underestimation of SIRs. The bias in the SIRs due to the use of general population rates, which included men with prostate cancer, to estimate expected numbers of thromboembolic disease was found to be negligible. The effect of treatment choice on the results should be small, since both history of thromboembolic disease and stage of disease were adjusted for. However, the above notwithstanding, there might be some residual bias that cannot be accounted for. For example, choice of endocrine treatment is likely to be related to comorbidity, suggesting that there could be a selection bias. However, as suggested by Miettinen,^41^ the physician or patient's choice of endocrine treatment primarily constitutes a confounder for the study of the intended effect (palliative treatment for prostate cancer), but not for the study of side-effects such as thromboembolic disease. This is because at the time the data were collected, the literature did not suggest a strong association between endocrine treatment and cardiovascular side-effects, thus it was not standard clinical practice to take thromboembolic disease history into account when initiating treatment. The diagnosis of prostate cancer itself can also bias the results, because these men receive more intensive medical care (eg, increased number of clinical visits), and are therefore more likely to be diagnosed with a thromboembolic disease event when it occurs. Furthermore, the combination of prostate cancer, especially advanced disease, with thromboembolic disease might strengthen the indication for hospitalisation, therefore biasing the SIR estimates upwards. Based on the Swedish Drug Registry, it was shown that it takes about 1 month before men with prostate cancer start taking their endocrine treatment (Stattin P, unpublished). The effect of delayed start of treatment was assessed with a sensitivity analysis excluding cardiovascular disease events that occurred within 1 month of the prostate cancer diagnosis, and showed almost no change in SIR estimates (data not shown). Furthermore, an unknown proportion of men treated curatively, on surveillance, or on anti-androgens, subsequently changed to GNRH agonists, which could dilute a true difference in risk between anti-androgens and GNRH agonists. We had no information about smoking habits, diabetes, body-mass index, or hypertension, but none of these factors are strongly associated with prostate-cancer risk, and are therefore unlikely to explain the current findings.^42^ No information was available on history of comorbidities other than circulatory diseases.

Our findings indicate that it is important to consider thromboembolic side-effects when treating patients with prostate cancer, especially those who require endocrine treatment. Higher risks for thromboembolic disease were also noted for younger men and men with metastatic disease. Risk patterns for thromboembolic disease that differ according to prostate-cancer treatment, age, and tumour stage, are probably explained by the physiological effects of prostate cancer, treatments for prostate cancer, and the factors taken into consideration when selecting these treatments.

## Web Extra Material

Supplementary webappendix
