# Supplementary material for: Risk of thromboembolic diseases in men with prostate cancer: results from the population-based PCBaSe Sweden
Source: Lancet Oncol. 2010 May 11;11(5):450–8. doi: 10.1016/S1470-2045(10)70038-3 (PMC2861771; doi:10.1016/S1470-2045(10)70038-3)
Supplement: Supplementary webappendix [file mmc1.pdf]

THE LANCET Oncology

Supplementary webappendix

This webappendix formed part of the original submission and has been peer reviewed. We post it as supplied by the authors.

Supplement to: Van Hemelrijck M, Adolfsson J, Garmo H, et al. Risk of thromboembolic diseases in men with prostate cancer: results from the population-based PCBaSe Sweden. *Lancet Oncol* 2010; published online April 14. DOI:10.1016/S1470-2045(10)70038-3.

Risk of thromboembolic disease in men with prostate cancer: Methods used to calculate SIRs

Using PCBaSE Sweden as our study cohort, we analyzed the relation between different treatments for prostate cancer, especially different types of endocrine treatment (ET), and groups of thromboembolic disease: deep-venous thrombosis (ICD-10: I80-82), deep-venous thrombosis (ICD-10: I26), and arterial embolism (ICD-10: K55, I74).

PCBaSE Sweden is based on the entire Swedish population, allowing for analysis of the risk of developing heart disease by use of standardized incidence ratios (SIR). These measurements made it possible to compare findings in the prostate cancer patient group with findings in a standard population such as the total Swedish population. The following sections will explain in more detail how the SIRs were calculated. Deep-venous thrombosis is used as an example, but the same methods were applied for the other thromboembolic diseases studied.

The SIR was defined as the ratio of observed numbers of deep-venous thrombosis to the expected numbers and the calculation of these observed and expected numbers is explained below. All numbers were based on the first event of deep-venous thrombosis after prostate cancer diagnosis.

The observed numbers of deep-venous thrombosis

The observed numbers of deep-venous thrombosis were counted among prostate cancer patients registered in PCBaSe Sweden based on treatment prescribed at time of diagnosis: watchful waiting, treatment with curative intent, and ET. The numbers of deep-venous thrombosis were counted by age and calendar-time categories and were found in the Hospital Discharge Register, which registered non-fatal events of heart disease between 1987 and 2007. However, counting the number of deep-venous thrombosis was more complicated in these analyses, as not only age and calendar-time, but also history of deep-venous thrombosis was taken into account. When calculating SIRs, it was important to compare prostate cancer patients with a particular history of thromboembolic disease to men in the total Swedish population with a similar history of thromboembolic disease.

Observed numbers of deep-venous thrombosis for men without history of thromboembolic disease

The numbers of deep-venous thrombosis among men without history of deep-venous thrombosis were only counted by age and calendar-time categories. Age was divided into 5-year age categories starting at age 20 and ending at age 100 for the incidence calculations. Calendar-time was split per year from 1997 to 2007.

Observed numbers of deep-venous thrombosis for men with history of thromboembolic disease

To take history of deep-venous thrombosis into account, numbers of deep-venous thrombosis were counted by age, calendar-time, and history of thromboembolic disease categories. We illustrate the concept of categories by age, calendar-time, and history of deep-venous thrombosis, by introducing the Lexis diagram. The Lexis diagram is a graphical representation of deep-venous thrombosis incidence in a coordinate system with calendar-time as abscissa and age as ordinate. Individuals are represented by line segments of slope 1 joining (time, age) at study entry. Events are represented by points in this diagram (1). The figure shows a Lexis diagram where patients were followed up between 1997 and 2007. The red triangles indicate the event of interest, in this case first deep-venous thrombosis after study entry. The black lines represent the follow-up time for each individual.

In order to account for thromboembolic disease history, numbers of deep-venous thrombosis were also counted by categories of previous numbers of thromboembolic diseases, as well as by time between prostate cancer diagnosis and last thromboembolic disease. This time was split up as follows: <0.083, 0.083-0.25, 0.25-0.5, 0.5-1.5, 1.5-4, >4 years, and the number of previous thromboembolic disease was divided into the following categories: 1, 2, 3+.

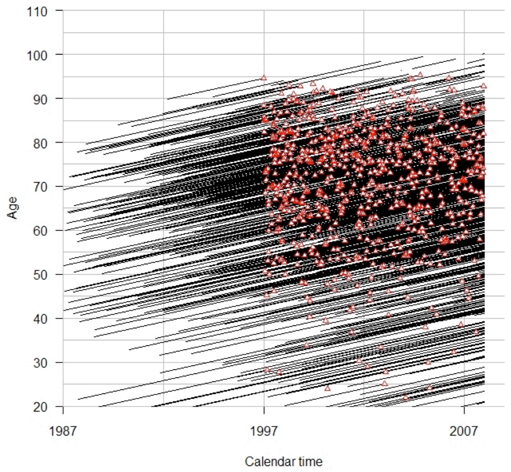

Figure: Example of a Lexis diagram.

Calculation of the expected numbers of deep-venous thrombosis
When calculating the expected numbers of deep-venous thrombosis, both the Hospital Discharge Register and the Register of the Total Population (standard population) were used (2, 3). The expected numbers of deep-venous thrombosis were calculated by multiplying the time of follow-up with the corresponding age- and period-specific incidence rates. The time of follow-up was taken from the observed numbers, i.e. the line segments of the corresponding Lexis diagrams in the prostate cancer patients. The age- and period-specific incidence rates were defined as the number of cases in the standard population divided by the corresponding person-time in this standard population. The categories used to calculate the numbers and person-time in the standard population are explained below.

Expected incidence for men without history of thromboembolic disease
As mentioned, the expected incidence was calculated by dividing the number of cases in the standard population by the corresponding person-time in this population (taken from the Register of the Total Population which is based on the number of people in Sweden each year on December 31st). Since this group did not have a history of thromboembolic disease, the expected numbers were only counted for categories of age and calendar-time. Age was divided into 5-year age categories starting at age 20 and ending at age 100. Calendar-time was split per year from 1997 to 2007. The expected number of events of deep-venous thrombosis was then calculated by multiplying the incidences of deep-venous thrombosis in the background population with the corresponding person-time observed for prostate cancer patients without history of deep-venous thrombosis.

Expected incidence for men with history of thromboembolic disease
When calculating the expected incidence of deep-venous thrombosis for men with history of thromboembolic disease, we had to count the events of deep-venous thrombosis in the standard population by categories of age, calendar-time, number of previous thromboembolic disease, and time since last thromboembolic disease. We used the following age and calendar-time groups: age (<50, 50-59, 60-69, 70-79, 80-89, 90+ years) and calendar-time (1997-2002 and 2003-2007). The time since last deep-venous thrombosis was split up as follows: <0.083, 0.083-0.25, 0.25-0.5, 0.5-1.5, 1.5-4, >4 years, and the number of previous deep-venous thrombosis was divided into the following categories: 1, 2, 3+. The expected numbers of events of deep-venous thrombosis were calculated by multiplying the incidences of deep-venous thrombosis in the background population with the corresponding person-time observed for prostate cancer patients with history of deep-venous thrombosis.

The 95% confidence intervals for the SIRs were estimated by assuming that the observed cases had a Poisson distribution using Byar's normal approximation (4, 5). Poisson Regression was used to adjust the SIRs and SMRs for cancer stage, history of thromboembolic disease, and SES. Finally, absolute risk increases by different types of thromboembolic disease and prostate cancer treatments were calculated and sensitivity analyses were conducted to test the assumption of intention-to-treat. Statistical analyses were performed with Statistical Analysis Systems (SAS) release 9.1.3 (SAS Institute, Cary, NC) and R version 2.7.2 (R Foundation for Statistical Computing, Wien, Austria).

References

1. Keiding N. Statistical inference in the Lexis diagram. Philosophical Transactions: Physical Sciences and Engineering1990;332(1627):23.

2. Central Bureau for Statistics. Statistics Sweden. Stockholm, Sweden2008; Available from: http://www.scb.se/.

3. Statistics in the Areas of Health and Medical Care [database on the Internet]2007. Available from: http://www.socialstyrelsen.se/en/Statistics/Statistical_databases.htm.

4. Breslow N, Day N. Statistical methods in cancer research. Volume II - The design and analysis of cohort studies. IARC Sci Publ1987;82:406.

5. Zar N, Garmo H, Holmberg L, Hellman P. Risk of second primary malignancies and causes of death in patients with adenocarcinoma and carcinoid of the small intestine. Eur J Cancer2008 Mar;44(5):718-25.
